# Plant food-derived antioxidant nutrients in neuroprotection against chemotherapy-induced neurotoxicity: from molecular mechanisms to clinical applications

**DOI:** 10.3389/fnut.2025.1667421

**Published:** 2025-12-05

**Authors:** Weizhong Li, Dan Yang, Zihan Zhang, Meizi Wang, Senlei Xu, Ruiyang Jiang, Jin Chen, Chaokui Wu, Jun Qian

**Affiliations:** 1 Department of Oncology, Affiliated Hospital of Nanjing University of Chinese Medicine, Nanjing, China; 2 Department of Obstetrics, Hospital of Chengdu University of Traditional Chinese Medicine, Chengdu, China; 3 Nanjing University of Chinese Medicine, Nanjing, China; 4 The First Affiliated Hospital of Guangzhou University of Chinese Medicine (Chongqing Beibei District Hospital of Traditional Chinese Medicine), Chongqing, China

**Keywords:** chemotherapy-induced peripheral neuropathy, plant-derived antioxidants, neuroprotection, curcumin, green tea, oxidative stress

## Abstract

Chemotherapy-induced peripheral neuropathy (CIPN) is one of the most challenging dose-limiting toxicities in contemporary oncology, affecting 19–85% of patients receiving neurotoxic chemotherapy agents. The pathophysiological mechanisms of CIPN are complex, involving multiple interconnected processes including oxidative stress, neuroinflammation, neurotrophic factor depletion, and mitochondrial dysfunction. Plant-derived antioxidant nutrients have emerged as promising candidates for CIPN prevention due to their unique multi-target neuroprotective capabilities. This comprehensive review systematically analyzes the molecular protective mechanisms of plant-derived nutrients, evaluates existing clinical evidence, and discusses practical application strategies. The focus is on the neuroprotective effects of curcumin, green tea catechins, vitamin E, and other plant compounds. Evidence indicates that these compounds exert protective effects through activation of endogenous antioxidant systems, modulation of inflammatory pathways, enhancement of neurotrophic factors, and protection of mitochondrial function. While clinical evidence is still accumulating, preliminary studies show encouraging results, providing a scientific basis for developing plant food-based CIPN prevention strategies.

## 1 Introduction

Chemotherapy-induced peripheral neuropathy (CIPN) is a common and debilitating dose-limiting toxicity, affecting 19–85% of patients treated with neurotoxic agents (1–3). Its sensorimotor symptoms—including numbness, tingling, and burning pain—often force dose reductions or discontinuation, compromising cancer outcomes (4, 5). Current therapies offer only modest symptom relief and fail to address the underlying mechanisms (6, 7) (Table 1).

**Table 1:** Summary of chemotherapy-induced neurotoxicity mechanisms by drug class.

Drug class	Representative agents	Primary neurotoxic mechanisms	Clinical manifestations	Affected nerve types
Platinum compounds	Cisplatin, Oxaliplatin	Direct DNA crosslink formation; Mitochondrial dysfunction; Rapid glutathione depletion; Excessive ROS generation	Numbness, tingling, burning pain in stocking-glove distribution	Predominantly sensory nerves
Taxanes	Paclitaxel, Docetaxel	Microtubule disruption; Impaired axonal transport; Antioxidant enzyme transport disorder; Distal oxidative stress hotspots	Length-dependent neuropathy with predominant distal symptoms	Primarily sensory nerves
Anthracyclines	Doxorubicin, Daunorubicin	Direct mitochondrial electron transport chain targeting; Superoxide and hydrogen peroxide generation; Impaired ATP-dependent antioxidant regeneration	Sensorimotor symptoms	Sensory and motor nerves
Vinca alkaloids	Vincristine	Microtubule function disruption; Axonal transport disorder; Neurotrophic factor depletion	Sensorimotor neuropathy	Sensory and motor nerves

The pathogenesis of CIPN is multifactorial, involving oxidative stress, neuroinflammation, neurotrophic factor depletion, and mitochondrial dysfunction (6–10). These convergent processes overwhelm the limited regenerative capacity of the peripheral nervous system, indicating that effective interventions must target multiple pathways simultaneously rather than isolated mechanisms (11).

Plant-derived foods have emerged as promising candidates for CIPN prevention due to their unique multi-target antioxidant properties. Their bioactive compounds can alleviate oxidative stress, modulate inflammatory cascades, preserve mitochondrial integrity, and enhance neurotrophic signaling, while offering safety, accessibility, and broad patient acceptance (12, 13). Advances in precision nutrition and nutrigenomics further expand their potential by enabling personalized interventions tailored to genetic and metabolic profiles (14–16).

This comprehensive review examines the current state of knowledge regarding plant food-derived nutrients in CIPN prevention and management. We analyze molecular mechanisms, evaluate clinical evidence, and discuss practical applications while identifying key research priorities for clinical translation.

## 2 Pathophysiological foundations of chemotherapy-induced neurotoxicity

The development of CIPN involves multiple convergent mechanisms that fundamentally alter the neurobiological landscape of peripheral nervous system function. These mechanisms arise from direct chemotherapy-induced cellular damage but subsequently evolve into self-perpetuating cycles of dysfunction and impaired repair (6–8, 17). Understanding these interconnected pathways provides essential context for evaluating how plant food-derived nutrients can effectively intervene in disease progression and support neural recovery (11, 18, 19).

### 2.1 Oxidative stress and cellular antioxidant depletion

**Figure 1:**
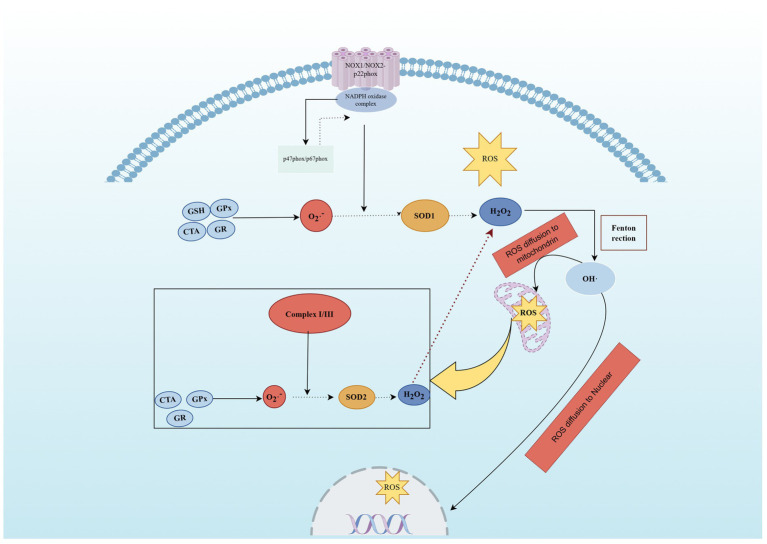
Chemotherapy exposure generates reactive oxygen species (ROS) via nicotinamide adenine dinucleotide phosphate (NADPH) oxidase and mitochondrial dysfunction, overwhelming antioxidant systems including glutathione (GSH), superoxide dismutase (SOD), and catalase. Abbreviations: ROS, reactive oxygen species; NADPH, nicotinamide adenine dinucleotide phosphate; GSH, glutathione; SOD, superoxide dismutase. Created with Figdraw (figdraw.com).

The peripheral nervous system demonstrates exceptional vulnerability to chemotherapy-induced oxidative damage due to its unique structural and metabolic characteristics, including long axonal transport distances, high polyunsaturated fatty acid content in neural membranes as major targets for lipid peroxidation, and weaker blood–nerve barrier protection compared with central structures (9, 20–23). Chemotherapy agents initiate oxidative stress through diverse pathways that collectively overwhelm cellular antioxidant defenses, with platinum-based compounds generating excessive reactive oxygen species through direct DNA interaction and mitochondrial dysfunction while rapidly depleting glutathione stores that serve as the primary intracellular antioxidant defense system (6, 24) (Figure 1).

Taxane agents contribute through distinct mechanisms involving microtubule disruption and impaired axonal transport of antioxidant enzymes and protective molecules. This creates localized oxidative stress hotspots in distal nerve regions, explaining the characteristic length-dependent pattern of taxane-induced neuropathy (9, 25). Anthracycline compounds directly target mitochondrial electron transport chains, generating superoxide radicals and hydrogen peroxide while simultaneously compromising ATP-dependent antioxidant regeneration systems. This creates vicious cycles where mitochondrial damage produces more oxidants that further impair cellular function and perpetuate neuronal dysfunction (24, 26).

The temporal dynamics of chemotherapy-induced oxidative stress involve both acute phases occurring during and immediately after treatment administration, characterized by rapid antioxidant depletion and damage marker accumulation, and chronic phases persisting long after treatment completion, driven by sustained mitochondrial dysfunction, impaired antioxidant enzyme expression, and ongoing inflammatory processes (6, 27). This persistent oxidative imbalance contributes significantly to the chronic nature of CIPN and its resistance to conventional therapeutic approaches (7).

### 2.2 Neuroinflammatory networks and immune system activation

Neuroinflammation represents a critical pathophysiological mechanism that amplifies and perpetuates neural damage beyond the direct cytotoxic effects of chemotherapy treatment through complex interactions between immune cells, neural tissue, and inflammatory mediators that create self-reinforcing cycles of damage and impaired repair (10, 28). The inflammatory cascade begins with chemotherapy-induced tissue damage that releases damage-associated molecular patterns, including high mobility group box 1, ATP, and DNA fragments, which activate pattern recognition receptors on resident macrophages and Schwann cells that respond by producing pro-inflammatory cytokines including tumor necrosis factor-alpha, interleukin-1 beta, and interleukin-6 (28–30).

These inflammatory mediators recruit additional immune cells and disrupt the blood-nerve barrier, allowing systemic inflammatory mediators to enter peripheral nerve tissue and further exacerbate local inflammation while creating a permissive environment for ongoing neural damage (29, 31–33). Nuclear factor-kappa B serves as a central mediator of chemotherapy-induced neuroinflammation, coordinating the expression of multiple pro-inflammatory genes in response to cellular stress and damage through multiple activation pathways including oxidative stress, DNA damage responses, and cytokine signaling cascades (28, 34).

Once activated, nuclear factor-kappa B translocates to the nucleus and initiates transcription of genes encoding inflammatory cytokines, chemokines, and enzymes such as cyclooxygenase-2 and inducible nitric oxide synthase that perpetuate inflammatory processes and directly damage neurons while impairing neurotrophic factor signaling and axonal regeneration (28, 34). Critically, chemotherapy-induced neuroinflammation often persists long after treatment completion, contributing to the chronic nature of CIPN and representing a potential therapeutic target for interventions aimed at breaking the cycle of ongoing inflammation and neural dysfunction.

### 2.3 Neurotrophic factor dysregulation and regenerative capacity impairment

The depletion of neurotrophic factors represents a crucial mechanism contributing to CIPN development and progression, as these essential proteins for neural survival, growth, differentiation, and synaptic plasticity demonstrate marked reductions during chemotherapy treatment through both direct suppression of gene expression by chemotherapy agents and indirect consequences of inflammation and oxidative stress that create a hostile environment for neurotrophic factor synthesis and signaling (6, 8, 35–38). Brain-derived neurotrophic factor and nerve growth factor, which are particularly important for maintaining sensory neurons predominantly affected in CIPN, show dramatic decreases that not only impair neuronal survival and increase susceptibility to chemotherapy-induced damage but also compromise the regenerative capacity of peripheral nerves, contributing to the persistent nature of CIPN and limiting recovery potential even after treatment completion (17, 39–43).

This neurotrophic factor deficiency creates a cascade of dysfunction that extends from direct effects on neuronal survival and growth to broader impacts on synaptic plasticity, pain processing, and neural adaptation mechanisms that are essential for maintaining normal sensory function (44–47). The spatial and temporal patterns of neurotrophic factor depletion correlate closely with the characteristic distal-to-proximal progression of neuropathy symptoms, suggesting that restoration of neurotrophic support represents a critical therapeutic target for both prevention and treatment of CIPN (48–50).

### 2.4 Mitochondrial bioenergetic crisis and cellular energy metabolism

**Figure 2:**
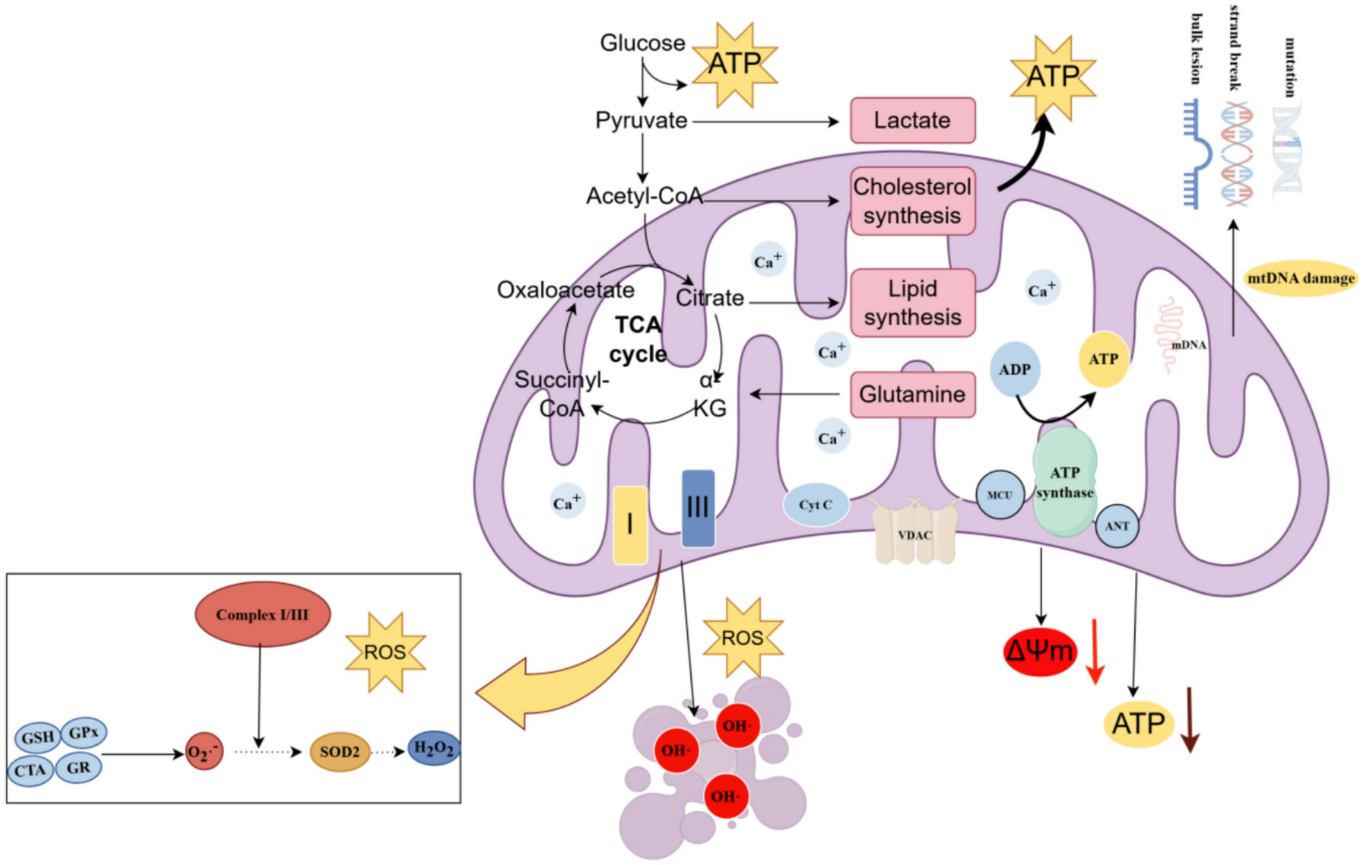
Chemotherapy disrupts mitochondrial electron transport chain complexes and the tricarboxylic acid (TCA) cycle, leading to mitochondrial DNA (mtDNA) damage, adenosine triphosphate (ATP) depletion, calcium (Ca^2+^) overload, and loss of mitochondrial membrane potential (ΔΨm). Abbreviations: TCA, tricarboxylic acid; mtDNA, mitochondrial DNA; ATP, adenosine triphosphate;ΔΨm, mitochondrial membrane potential. Created with Figdraw (figdraw.com).

Mitochondrial dysfunction emerges as a fundamental mechanism underlying chemotherapy-induced neurotoxicity, as these organelles serve as both primary targets of chemotherapy-induced damage and critical determinants of neuronal survival and function, with the peripheral nervous system’s exceptionally high energy demands making it particularly vulnerable to mitochondrial impairment since neurons rely heavily on oxidative phosphorylation for ATP generation and lack the glycolytic capacity to compensate for mitochondrial dysfunction (39, 51–54). Chemotherapy agents affect mitochondrial function through multiple interconnected mechanisms, including direct accumulation in mitochondria with formation of DNA crosslinks that impair replication and transcription, disruption of mitochondrial dynamics and transport processes, and direct inhibition of electron transport complexes, leading to ATP depletion and enhanced reactive oxygen species production that triggers apoptotic pathways and neuronal death (55–61) (Figure 2).

**Figure 3:**
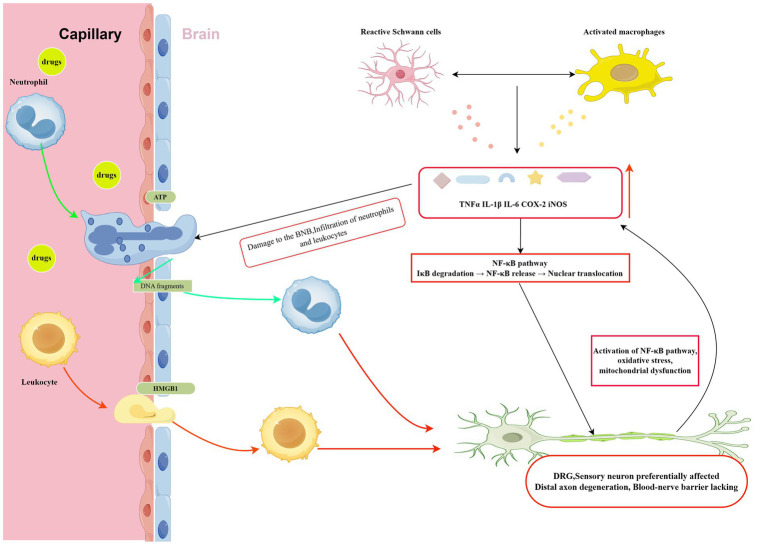
Chemotherapy damages the blood–nerve barrier, releasing damage-associated molecular patterns (DAMPs) such as high-mobility group box 1 (HMGB1), which activate Schwann cells and macrophages and induce nuclear factor-kappa B (NF-κB)-mediated cytokine production including tumor necrosis factor-alpha (TNF-α), interleukin-1 beta (IL-1β), and interleukin-6 (IL-6), ultimately injuring dorsal root ganglion (DRG) neurons. Abbreviations: DAMPs, damage-associated molecular patterns; HMGB1, high-mobility group box 1; NF-κB, nuclear factor-kappa B; TNF-α, tumor necrosis factor-alpha; IL-1β, interleukin-1 beta; IL-6, interleukin-6; DRG, dorsal root ganglion. Created with Figdraw (figdraw.com).

The bioenergetic crisis particularly affects distal nerve segments where energy demands are highest and mitochondrial transport distances are greatest, explaining the characteristic length-dependent pattern of chemotherapy-induced neuropathy and its predilection for sensory neurons that have particularly long axons and high metabolic demands (62–64) (Figure 3). Mitochondrial dysfunction also impairs the cell’s ability to maintain calcium homeostasis, support axonal transport, and synthesize essential proteins and lipids needed for membrane maintenance and repair, creating a cascade of cellular dysfunction that extends far beyond simple energy depletion to encompass fundamental aspects of neuronal structure and function (65–68).

## 3 Plant food Neuroprotective mechanisms: molecular targets and pathways

### 3.1 Endogenous antioxidant system activation

Plant polyphenolic compounds provide neuroprotection primarily through activation of the nuclear factor erythroid 2-related factor 2/antioxidant response element (Nrf2/ARE) pathway (69, 70) (Table 2). This pathway serves as the master regulator of cellular antioxidant defenses (71). Under normal conditions, Nrf2 remains sequestered in the cytoplasm by Kelch-like ECH-associated protein 1 (Keap1) (72). Keap1 facilitates rapid Nrf2 degradation through the ubiquitin-proteasome system (73).

**Table 2:** Comparative analysis of plant food compounds for neuroprotection.

Compound	Primary food sources	Core protective mechanisms	Molecular targets
Curcumin	Turmeric, curry powder	Nrf2/ARE pathway activation; NF-κB signaling inhibition; α7 nicotinic receptor modulation	Keap1, IκB kinase, α7 nAChR
EGCG	Green tea	Mitochondrial protection; Anti-inflammatory effects; Neurotrophic factor enhancement	Mitochondrial electron transport chain, NF-κB pathway
Resveratrol	Grapes, berries	Sirtuin 1 pathway activation; NF-κB deacetylation; Anti-inflammatory action	SIRT1, NF-κB subunits
Vitamin E	Nuts, seeds	Lipid peroxidation protection; Membrane stabilization; Antioxidant enzyme support	Cell membrane phospholipids
α-Lipoic acid	Spinach, broccoli	Mitochondrial antioxidant action; PGC-1α activation; Enzyme cofactor functions	Mitochondrial enzyme complexes, PGC-1α
Soy isoflavones	Soy products	Selective estrogen receptor modulation; BDNF gene transcription activation; Neurotrophic factor enhancement	ERβ, BDNF promoter

Plant polyphenolic compounds disrupt this regulatory interaction through multiple mechanisms. They form direct covalent modifications of Keap1 cysteine residues (70, 74). They also promote phosphorylation of Nrf2 by upstream kinases and create competitive inhibition of protein–protein binding interactions (75, 76). Once released from Keap1-mediated repression, Nrf2 translocates to the nucleus (70, 74). There it forms heterodimers with small Maf proteins and binds to antioxidant response element sequences (77, 78).

This transcriptional activation upregulates numerous cytoprotective enzymes. These collectively enhance cellular resistance to oxidative stress and toxic insults (79, 80). Key enzymes include heme oxygenase-1, which catalyzes heme degradation to produce powerful antioxidant and anti-inflammatory products (81, 82). NAD(P)H: quinone oxidoreductase 1 functions as a two-electron reductase preventing quinone participation in redox cycling reactions (83, 84). Glutathione S-transferases facilitate glutathione conjugation to electrophilic compounds for detoxification and elimination (85, 86).

Curcumin exemplifies the potent Nrf2-activating potential of plant polyphenols. Its diferuloylmethane structure contains multiple electrophilic sites that form covalent adducts with Keap1 cysteine residues (87, 88). This leads to conformational changes that disrupt Nrf2 binding. Curcumin simultaneously activates upstream kinases including protein kinase C and mitogen-activated protein kinases. These phosphorylate Nrf2 and promote its nuclear translocation (89–91). This mechanism is particularly relevant in CIPN, as Nrf2-driven upregulation of glutathione and phase II detoxification enzymes directly counteracts platinum-induced glutathione depletion, while enhancement of antioxidant enzymes such as SOD and catalase mitigates the distal oxidative stress hotspots characteristic of taxane-induced neuropathy.

### 3.2 Anti-inflammatory pathway modulation

Plant polyphenols demonstrate potent anti-inflammatory activity through inhibition of nuclear factor-kappa B signaling (92). This represents the key transcriptional pathway coordinating inflammatory gene expression in response to cellular stress (93). Curcumin functions as a particularly effective nuclear factor-kappa B inhibitor. It directly binds to nuclear factor-kappa B subunits, preventing their nuclear translocation and DNA binding (94). It also inhibits IκB kinase activity, preventing phosphorylation and degradation of IκB proteins (95).

The dual action of curcumin creates synergistic protective effects particularly relevant to chemotherapy-induced neuroinflammation prevention. It simultaneously activates anti-inflammatory Nrf2 pathways while inhibiting pro-inflammatory nuclear factor-kappa B signaling (96–98). This mechanistic duality explains curcumin’s demonstrated efficacy in reducing inflammatory cytokine expression and preserving nerve function in experimental neuropathy models (99–101).

Resveratrol from grapes and berries demonstrates anti-inflammatory effects through sirtuin 1 pathway activation (102). This deacetylates and inactivates nuclear factor-kappa B subunits while promoting cellular stress resistance (103, 104). Enhanced DNA repair and metabolic optimization provide sustained anti-inflammatory effects that promote cellular adaptation to stress (105–109).

Green tea catechins provide complementary anti-inflammatory activity through multiple pathways. These include direct inhibition of inflammatory enzymes such as cyclooxygenase-2 and lipoxygenase (110, 111). They also suppress inflammatory cytokine production and modulate immune cell activation patterns (112, 113). These compounds enhance the production of anti-inflammatory mediators and promote active resolution of inflammatory processes. This anti-inflammatory action directly suppresses the NF-κB–mediated cytokine cascades that drive persistent neuroinflammation and amplify chemotherapy-induced neural damage.

### 3.3 Neurotrophic factor enhancement systems

Plant foods contain numerous compounds that specifically support neurotrophic factor synthesis and signaling. These provide essential nutritional support for neural survival, growth, and repair processes (114–117). Flavonoid compounds from various plant sources demonstrate the ability to cross the blood–brain barrier (116). They directly stimulate brain-derived neurotrophic factor synthesis in neural tissue through activation of cAMP response element-binding protein pathways (115, 116, 118, 119). This transcriptional activation drives brain-derived neurotrophic factor gene transcription via phosphorylation-dependent mechanisms. The result is increased brain-derived neurotrophic factor expression that enhances neuronal survival. It also promotes axonal growth and supports synaptic plasticity essential for neural adaptation and recovery (120–123).

Soy isoflavones, particularly genistein and daidzein, demonstrate unique neurotrophic properties (124–126). These occur through their selective estrogen receptor modulator activities (127–129). They bind preferentially to estrogen receptor beta, which is highly expressed in neural tissue (130, 131). This promotes brain-derived neurotrophic factor gene transcription via estrogen response elements (132–135). The result is increased brain-derived neurotrophic factor protein synthesis and release (133, 135–137).

The neurotrophic effects of soy isoflavones extend beyond brain-derived neurotrophic factor modulation. They enhance nerve growth factor synthesis in Schwann cells, the primary supportive cells of peripheral nerves (138–140). They also promote expression of neurotrophin-3 and neurotrophin-4 that support different populations of sensory neurons (141). This makes these compounds particularly valuable for preventing and treating peripheral neuropathy. This neurotrophic support directly addresses the depletion of BDNF and NGF that limits peripheral nerve survival and regenerative capacity during chemotherapy.

### 3.4 Mitochondrial protection and bioenergetic enhancement

Plant foods provide mitochondrial protective nutrients that deliver essential bioenergetic support (142–145). These can prevent chemotherapy-induced mitochondrial damage and promote neural recovery through enhanced cellular energy production and reduced oxidative stress. α-Lipoic acid, found naturally in spinach, broccoli, and other green vegetables, functions as a unique mitochondrial antioxidant (146, 147).

This compound possesses both lipophilic and hydrophilic properties that allow comprehensive protection in both aqueous and lipid phases (148). It serves as an essential cofactor for mitochondrial enzyme complexes including pyruvate dehydrogenase and *α*-ketoglutarate dehydrogenase (149). These catalyze crucial steps in cellular energy production. α-Lipoic acid also functions as a potent antioxidant that directly scavenges reactive oxygen species while regenerating other antioxidants (150, 151).

The protective effects extend to activation of peroxisome proliferator-activated receptor gamma coactivator 1-alpha (PGC-1α). This serves as the master regulator of mitochondrial biogenesis (152, 153). PGC-1α activation promotes transcription of nuclear genes encoding mitochondrial proteins while enhancing mitochondrial DNA replication and protein synthesis (154, 155).

Coenzyme Q10, obtained from plant foods including spinach, cauliflower, and whole grains, serves as an essential component of the mitochondrial electron transport chain (156). It shuttles electrons between Complex I/II and Complex III while playing a crucial role in oxidative phosphorylation and ATP synthesis (157, 158). Coenzyme Q10 also functions as a powerful lipid-phase antioxidant that protects mitochondrial membranes from oxidative damage (158, 159).

Pyrroloquinoline quinone (PQQ), found in fermented soy products, green tea, and various fruits and vegetables, demonstrates unique mitochondrial protective properties (160). It functions as a redox cofactor for various enzymes while demonstrating potent antioxidant activity and mitochondrial biogenesis-promoting effects (161). PQQ can directly activate PGC-1α through cAMP/cAMP response element-binding protein signaling pathways. This leads to enhanced mitochondrial biogenesis and improved cellular bioenergetic capacity (162). These protective effects directly oppose the mitochondrial bioenergetic failure caused by platinum- and taxane-induced impairment of oxidative phosphorylation, calcium homeostasis, and axonal energy supply.

## 4 Clinical evidence landscape for plant food interventions

The translation of preclinical neuroprotective mechanisms into clinical applications has generated substantial research interest and a growing body of evidence. However, direct clinical evidence for plant food interventions in chemotherapy-induced peripheral neuropathy prevention remains limited compared to the extensive and consistently positive preclinical foundation (163–165). This highlights the critical need for well-designed clinical trials that can bridge the gap between mechanistic understanding and therapeutic application while addressing the unique challenges associated with studying complex, multi-component dietary interventions in cancer patient populations.

### 4.1 Turmeric and curcumin: evidence chain validation

Curcumin is the plant-derived compound with the most comprehensive validation for CIPN prevention, anchored by a landmark Level I pediatric randomized trial and further supported by cross-disease and mechanistic studies (166). This double-blind study enrolled 141 children aged 5–15 years with acute lymphoblastic leukemia receiving vincristine-based chemotherapy protocols. Patients randomized to oral curcumin supplementation at 3 mg/kg twice daily for 3 months demonstrated significant reduction in vincristine-induced peripheral neuropathy incidence from 70.0% in the placebo group to 39.4% in the treatment group (Table 3). Beyond incidence reduction, curcumin treatment resulted in significantly improved Total Neuropathy Score-Pediatric Vincristine (TNS-PV) evaluations and reduced motor nerve electrophysiological abnormalities, providing objective validation of genuine neuroprotective effects.

**Table 3:** Clinical evidence summary for plant food interventions.

Study Compound	Study Design	Participants	Intervention Protocol	Primary Outcomes	Efficacy	Evidence Level
Curcumin	Double-blind RCT	141 children aged 5–15 with ALL	3 mg/kg twice daily, 3 months	Neuropathy incidence: 39.4% vs. 70.0%; Significant TNS-PV improvement	Highly effective	Level I
Nano-curcumin	RCT	Type 2 diabetes sensorimotor polyneuropathy patients	80 mg once daily, 8 weeks	Reduced HbA1c and fasting glucose; Improved total neuropathy score	Effective	Level II
Green tea extract	RCT	Mild–moderate diabetic peripheral neuropathy patients	16 weeks supplementation	Reduced VAS pain scores; Improved TCSS symptoms; Better vibration thresholds	Effective	Level II
Green tea polyphenols	Preliminary clinical study	Liver cancer chemotherapy patients	474 mg polyphenol tablets daily	Significantly reduced treatment-induced oxidative stress	Effective	Level III
Vitamin E	RCT	108 cisplatin chemotherapy patients	400 mg daily	Lower peripheral neuropathy incidence; Improved nerve conduction parameters	Effective	Level II
Omega-3 fatty acids	Double-blind RCT	Breast cancer paclitaxel patients	640 mg three times daily	70% neuropathy-free vs. 40.7% (placebo)	Highly effective	Level I

Cross-disease validation arises from neuropathies that share convergent mechanisms. In patients with type 2 diabetes-associated sensorimotor polyneuropathy, nano-curcumin 80 mg once daily for 8 weeks significantly reduced HbA1c and fasting plasma glucose while improving the total neuropathy score, reflexes and skin temperature (167). Although median nerve conduction velocity was not assessed, the concordant amelioration of clinical signs and symptoms provides indirect yet compelling support for curcumin’s capacity to counter peripheral nerve dysfunction via antioxidant and anti-inflammatory pathways. Such cross-condition efficacy strengthens the translational case for curcumin as a broad neuroprotective agent against disorders that share common pathophysiological drivers (168–170).

Extensive animal data establish curcumin’s pharmacological foundation across multiple chemotherapy-induced neuropathy models. In paclitaxel-induced peripheral neuropathy mice, dietary supplementation with 1.5% curcumin-phospholipid complex (Meriva®) completely blocked both mechanical and cold pain hypersensitivity (171). Mechanistic investigations revealed that this protection operates through the α7 nicotinic acetylcholine receptor (α7 nAChR) signaling axis, which suppresses spinal cord inflammatory responses. The specificity of this mechanism was definitively established through α7 nAChR gene knockout mice studies, where curcumin’s protective effects were completely eliminated. Administration of α7 nAChR receptor antagonists similarly abolished the neuroprotective benefits, confirming α7 nAChR as the critical molecular target.

Validation across different chemotherapy agents is robustly demonstrated in models of cisplatin-, vincristine-, and oxaliplatin-induced neuropathy. In oxaliplatin models, curcumin dose-dependently alleviates mechanical and cold hypersensitivity, improves both motor (MNCV) and sensory (SNCV) nerve conduction velocities, and repairs damaged spinal neurons. This protection is mechanistically linked to its ability to enhance endogenous antioxidant enzymes (SOD, GSH-Px, CAT) while reducing lipid peroxidation (MDA), and critically, to suppress neuroinflammation by inhibiting the activation of the NF-κB pathway and its downstream inflammatory cytokines (TNF-*α*, IL-1β, IL-6) (98). Similarly, in vincristine-induced neuropathy, advanced formulations like curcumin nano-emulsions significantly reduce cold and thermal hyperalgesia by boosting antioxidant (SOD, CAT) and anti-inflammatory (IL-10) markers, while downregulating NF-κB expression in the sciatic nerve (172). In cisplatin models, curcumin nanoparticles effectively counteract neurotoxicity by normalizing levels of lipid peroxidation, TNF-α, and caspase-3, and restoring depleted glutathione and Na+, K + -ATPase activity (173, 174). Collectively, these studies confirm that curcumin’s efficacy stems from its multi-target ability to mitigate oxidative stress, suppress the NF-κB-driven neuroinflammatory cascade, and preserve both the structure and function of peripheral nerves.

The curcumin evidence represents the most comprehensive clinical validation among plant-derived neuroprotective compounds, with the pediatric randomized controlled trial providing Level I evidence for efficacy, cross-condition validation demonstrating broader therapeutic potential, and robust preclinical data elucidating specific molecular mechanisms through α7 nAChR-Nrf2 dual pathway targeting. This evidence integration demonstrates completion of a comprehensive “clinical-preclinical-mechanistic” validation loop that positions curcumin as having achieved the most complete validation profile among plant-derived neuroprotective compounds.

### 4.2 Green tea: epidemiological and intervention evidence

Green tea catechins represent an emerging intervention with promising evidence from related neuropathies and ongoing clinical trials. Green tea is a widely consumed polyphenolic beverage with substantial neuroprotective potential (175–177). Green tea contains a complex mixture of bioactive compounds with catechins representing the predominant polyphenolic constituents. Epigallocatechin gallate (EGCG) comprises 30–60% of total catechins and demonstrates the most potent neuroprotective activities through its ability to cross the blood–brain barrier and accumulate in neural tissue (175, 178).

Robust epidemiological evidence provides a strong population-level basis for green tea’s neuroprotective effects. Large-scale, long-term observational studies consistently link green tea consumption with superior nervous system health, showing an inverse association with the risk of cognitive decline and neurodegenerative diseases such as Alzheimer’s and Parkinson’s disease (179–182). These real-world data offer a compelling rationale for investigating green tea as a targeted intervention.

Preclinical research provides clear mechanistic support for EGCG’s protective role against CIPN and related chemotherapy-induced toxicities. In mouse models of cisplatin-induced nephrotoxicity, a condition sharing oxidative stress pathways with CIPN, EGCG was shown to significantly mitigate tissue damage by protecting mitochondrial function, restoring electron transport chain activity, enhancing mitochondrial antioxidant enzymes (e.g., GPX and MnSOD), and suppressing inflammation via the NF-κB pathway (183–185). Similar protective effects against cisplatin-induced hepatotoxicity have also been observed when EGCG is combined with Coenzyme Q10 (186). Furthermore, demonstrating a direct neuro-regenerative potential, green tea polyphenols, particularly EGCG, have been shown to enhance nerve growth factor (NGF)-induced neurite outgrowth in cell culture models (187, 188).

While large-scale clinical trials for CIPN are still emerging, compelling evidence comes from studies on related neuropathies. A randomized controlled trial in patients with mild-to-moderate diabetic peripheral neuropathy (DPN), a condition with similar pathological mechanisms to CIPN, found that 16 weeks of green tea extract supplementation resulted in significant improvements compared to placebo (189). Specifically, patients experienced reductions in pain (Visual Analogue Scale, VAS), total symptoms (Total Symptom Score, TCSS), and vibration perception thresholds (VPT), with benefits becoming apparent as early as 8 weeks into treatment.

In the context of oncology, the evidence is moving from indirect support to direct validation. A preliminary clinical study in liver cancer patients undergoing chemotherapy found that daily supplementation with green tea polyphenol tablets (equivalent to ~474 mg of polyphenols) significantly reduced treatment-induced oxidative stress (190). More significantly, a frontier of clinical research has been opened with the initiation of a Phase 1/2 clinical trial (NCT06524609) specifically designed to evaluate a topical EGCG solution for the prevention and treatment of taxane-induced peripheral neuropathy (TIPN). The launch of this trial marks a critical step in translating EGCG’s potential into a viable clinical intervention for CIPN.

A particularly compelling aspect of EGCG is its dual functionality in cancer therapy: it not only protects normal tissues from chemotherapy-induced damage but can also enhance the cytotoxic effects of chemotherapy on cancer cells (191, 192). This chemosensitizing effect is achieved through multiple mechanisms. For instance, EGCG has been shown to enhance the efficacy of platinum-based drugs like cisplatin and oxaliplatin by inducing autophagy in colorectal cancer cells (193). Other studies have demonstrated that EGCG can increase cancer cell sensitivity to cisplatin by inhibiting the DNA repair enzyme ERCC1/XPF, or by upregulating the platinum drug transporter CTR1, thereby increasing intracellular drug accumulation in lung and ovarian cancer cells (194). This unique ability to both protect nerves and potentiate anti-cancer treatment makes EGCG a highly attractive candidate for adjuvant therapy.

### 4.3 Nuts and seeds: vitamin E clinical trial lessons

Despite promising mechanistic rationale, the clinical evidence for Vitamin E presents a more complex and sometimes contradictory picture. Evidence for vitamin E’s neuroprotective potential comes from multiple randomized trials with mixed results but important methodological insights (195–204). These studies highlight important considerations regarding supplement forms, dosing strategies, patient populations, and study methodologies that have significant implications for future research and clinical application.

The landmark positive study by Pace et al. randomized 108 patients receiving cisplatin-based chemotherapy to vitamin E supplementation at 400 mg daily or placebo. The study demonstrated significantly lower incidence of peripheral neuropathy in the vitamin E group compared to placebo with better preserved nerve conduction parameters and reduced symptom severity scores (197). However, larger subsequent trials have produced conflicting results that highlight the complexity of translating promising preliminary findings into consistent clinical benefits (200). These contradictory results highlight several important methodological considerations including the use of different vitamin E forms (synthetic *α*-tocopherol versus natural mixed tocopherols), varying dosing regimens, diverse patient populations with different baseline nutritional status, different chemotherapy agents with distinct mechanisms of neurotoxicity, and heterogeneous neuropathy assessment methods (195, 196, 205). Synthetic α-tocopherol supplementation may deplete or competitively inhibit *γ*-tocopherol and tocotrienols, thereby diminishing overall antioxidant defense, whereas whole-food sources provide these compounds in physiologic ratios with additional polyphenols, minerals, and unsaturated fatty acids that act synergistically to stabilize neuronal membranes and modulate inflammation (206–210).

The contradictory clinical evidence strongly emphasizes the importance of obtaining vitamin E from whole food sources rather than isolated synthetic supplements. Nuts and seeds provide vitamin E in natural ratios with other tocopherols and tocotrienols along with complementary antioxidants, minerals, and bioactive compounds that may enhance protective effects through synergistic interactions (211, 212). Natural vitamin E from food sources also provides more sustained tissue levels through gradual absorption compared to rapid absorption and clearance associated with high-dose supplement administration (213–216).

Plant-based omega-3 sources from walnuts, flaxseeds, and chia seeds contribute to anti-inflammatory neuroprotection through *α*-linolenic acid that serves as a precursor for longer-chain omega-3 fatty acids (217, 218). A double-blind RCT (NCT01049295) showed that 640 mg omega-3 TID during paclitaxel therapy kept 70% of breast-cancer patients neuropathy-free versus 40.7% with placebo, underscoring the protective potential of essential n-3 fatty acids (219).

### 4.4 Fruits and vegetables: carotenoid and anthocyanin evidence

For carotenoids and anthocyanins, clinical data remain limited, but preclinical and cognitive studies provide supportive evidence. Colorful fruits and vegetables provide carotenoids and anthocyanins with specialized neuroprotective properties that demonstrate significant promise in preclinical studies. Direct clinical evidence in chemotherapy-induced peripheral neuropathy remains limited, but supportive evidence from neurodegeneration studies suggests broader protective potential (220–222). Dark leafy greens provide lutein and zeaxanthin, xanthophyll carotenoids that cross the blood–brain barrier and accumulate in neural tissue (223–225).

A meta-analysis of 4,402 older adults demonstrated that carotenoid supplementation significantly improved global cognition (226); large cross-sectional datasets such as NHANES likewise associate higher intakes of *α*- and β-carotene, lutein and zeaxanthin with better performance in memory, processing speed and attention tests (227, 228). The mechanistic foundation for carotenoid neuroprotection stems from their unique molecular structure and tissue distribution patterns (229–231). These compounds demonstrate preferential accumulation in neural tissue regions with high metabolic activity and oxidative stress vulnerability. The conjugated double-bond systems characteristic of carotenoids provide exceptional capacity for singlet oxygen quenching and free radical neutralization, while their lipophilic nature enables integration into neural membrane phospholipids where they stabilize membrane architecture and protect against lipid peroxidation (230–233). This mechanism is particularly relevant to chemotherapy-induced neurotoxicity, where oxidative membrane damage represents a primary pathological process.

Berry anthocyanins represent another compelling class of neuroprotective compounds with emerging evidence for peripheral nerve protection (234). These deeply pigmented flavonoid compounds demonstrate sophisticated abilities to modulate neuroinflammatory cascades while simultaneously enhancing neurotrophic factor expression and supporting cellular repair mechanisms (234, 235). Preclinical studies have shown that anthocyanin-rich extracts from blueberries, blackberries, and other dark berries can reduce oxidative stress markers and inflammatory cytokine expression in neural tissue while promoting nerve growth factor synthesis (236, 237). The protective mechanisms of anthocyanins operate through multiple convergent pathways including potent nuclear factor-κB pathway inhibition, enhancement of brain-derived neurotrophic factor expression in neural tissue, and direct antioxidant effects that complement carotenoid protection through different molecular targets (236, 237).

## 5 Practical application of plant food neuroprotection

The convergence of mechanistic understanding and clinical evidence detailed in the preceding sections provides a compelling rationale for the clinical application of plant-based neuroprotective strategies. However, translating these research findings into effective, safe, and feasible dietary protocols for patients undergoing chemotherapy requires careful consideration. This section, therefore, aims to delineate evidence-based principles for the selection, preparation, and combination of neuroprotective plant foods, thereby bridging the gap between scientific knowledge and practical implementation in patient care.

### 5.1 Evidence-based food selection and prioritization

Given the varying levels of clinical validation across different plant foods, a tiered approach to food selection provides the most rational foundation for practical implementation.

Tier 1 foods include those with direct clinical evidence in neuropathy prevention, primarily turmeric-containing foods and green tea, which should form the foundation of any plant food neuroprotection strategy (163, 179, 238, 239) (Table 4). Daily consumption of turmeric-spiced foods prepared with fat and black pepper, along with 2–3 cups of green tea, provides clinically relevant doses of curcumin and EGCG with established safety profiles (239–241). An illustrative example of nutrient synergy is the combination of curcumin with piperine, a bioactive alkaloid from black pepper (242). Piperine inhibits hepatic and intestinal glucuronidation, thereby slowing curcumin metabolism and increasing its systemic exposure (243). This interaction provides a mechanistic rationale for pairing turmeric with black pepper in culinary and clinical contexts, underscoring that the form and combination of bioactive compounds can be as critical as their individual properties for achieving therapeutic efficacy.

**Table 4:** Evidence-based tiered food selection guidelines.

Evidence tier	Recommended foods	Specific sources	Preparation methods	Special considerations
Tier 1 (Direct clinical evidence)	Turmeric-spiced foods	Fresh turmeric, turmeric powder, curry dishes	Stewing, stir-frying with coconut or olive oil	Avoid on empty stomach; Monitor anticoagulant interactions
	Green tea	High-quality green tea leaves	Brew at 80 °C for 3–5 min	Avoid 1 h before meals; Consider caffeine content
Tier 2 (Strong preclinical evidence)	Nuts and seeds	Almonds, walnuts, sunflower seeds, flaxseeds	Light roasting or raw consumption	Choose natural unprocessed forms; Monitor allergic reactions
	Dark berries	Blueberries, blackberries, raspberries	Steam or heat preparation	Avoid raw during immunosuppression
	Dark leafy greens	Spinach, kale, broccoli	Thorough cooking	Ensure proper washing and cooking
	Soy products	Tofu, soy milk, miso	Adequate heat treatment	Choose organic non-GMO products
Tier 3 (Primarily mechanistic evidence)	Fermented foods	Sauerkraut, kimchi, fermented teas	Ensure proper fermentation	Use cautiously during immunosuppression
	Specific antioxidant vegetables	Carrots, bell peppers, tomatoes	Consume after cooking	Supplement diverse diet

Tier 2 foods encompass those with strong preclinical evidence and supportive clinical data from related conditions, including nuts and seeds rich in vitamin E and omega-3 fatty acids, colorful berries high in anthocyanins, and dark leafy greens providing multiple complementary compounds (244–249). These foods should be consumed regularly as part of a diverse dietary pattern that maximizes synergistic interactions while ensuring nutritional adequacy (246, 249–251).

Tier 3 foods include those with primarily mechanistic evidence but limited clinical validation, such as specific vegetables high in individual antioxidants or specialized fermented products (249, 252–254). While these foods may provide additional benefits, they should supplement rather than replace the evidence-based Tier 1 and 2 selections.

### 5.2 Addressing treatment-related dietary challenges

Chemotherapy treatment creates unique dietary challenges that require adaptive strategies to maintain neuroprotective food intake while accommodating treatment-related side effects and safety considerations (255). Nausea and altered taste perception, common with many chemotherapy regimens, can be addressed through strategic use of ginger-containing warm preparations and mild, room-temperature foods that minimize aversive sensory experiences (256, 257). Well-cooked vegetable soups combining leafy greens, root vegetables, and gentle spices provide concentrated nutrition in easily tolerated liquid forms that accommodate swallowing difficulties and reduced appetite.

Immunocompromised status during chemotherapy necessitates careful attention to food safety, requiring thorough cooking of all plant foods to eliminate potential pathogens (258, 259). Raw fruits and vegetables should be avoided, with emphasis on cooked preparations including steamed vegetables, baked fruits, and thoroughly heated plant-based soups and stews. Frozen vegetables and fruits can provide year-round access to neuroprotective compounds while offering convenience and safety advantages through processing that reduces microbial contamination risk.

Reduced appetite and early satiety require emphasis on nutrient-dense foods in smaller portions, with frequent consumption of small amounts of high-quality plant foods rather than attempting to maintain normal portion sizes (260–262). Concentrated sources such as cooked nut butters, well-cooked dried fruits, and warm herbal teas can provide meaningful neuroprotective compounds without requiring large food volumes. The incorporation of neuroprotective spices into familiar comfort foods can maintain nutritional goals while supporting psychological well-being during treatment.

Oral mucositis and swallowing difficulties, common with certain chemotherapy regimens, require modifications toward softer textures and neutral temperatures (263). Pureed vegetable soups, well-cooked mashed vegetables with olive oil, and lukewarm herbal teas provide gentle nutrition delivery that minimizes oral discomfort while maintaining neuroprotective compound intake. Room-temperature foods often provide better tolerance than hot or cold preparations during periods of oral sensitivity.

### 5.3 Long-term sustainability and patient-centered implementation

Successful long-term implementation requires integration of neuroprotective foods into sustainable dietary patterns that account for individual preferences, cultural backgrounds, and economic constraints (264, 265). Rather than prescriptive dietary protocols, flexible frameworks that allow for personal adaptation while maintaining core neuroprotective principles demonstrate superior adherence and long-term sustainability.

Cultural adaptation involves identifying traditional foods and preparation methods that naturally provide neuroprotective compounds, such as curry dishes in South Asian cuisines, tea ceremonies in East Asian cultures, and Mediterranean dietary patterns that emphasize olive oil, nuts, and vegetables. This approach respects cultural preferences while achieving therapeutic objectives through familiar and acceptable foods.

Economic considerations require emphasis on affordable, accessible plant foods that provide optimal neuroprotective value per unit cost. Dried legumes, seasonal vegetables, frozen berries, and basic spices often provide superior nutritional value compared to expensive supplements or exotic foods, while also being more widely available and culturally acceptable across diverse populations.

Patient education should focus on practical skills including food selection, preparation techniques, and meal planning that empowers individuals to make informed choices rather than relying on complex dietary protocols. Simple guidelines such as “eat colorful vegetables daily,” “include nuts or seeds with meals,” and “drink green tea regularly” provide actionable direction without overwhelming complexity.

## 6 Research limitations and future priorities

A critical limitation for the clinical translation of plant-derived polyphenols is their inherently low oral bioavailability (266). Compounds such as curcumin undergo rapid metabolism and systemic clearance, which significantly reduces their effective plasma and tissue concentrations (267). This pharmacokinetic barrier explains why the consistently positive results observed in preclinical models often fail to translate into equivalent efficacy in human trials using simple extracts (268). Advanced formulation strategies—including phospholipid complexes, nanoparticle encapsulation, and co-administration with piperine from black pepper—are therefore not merely technical details but essential requirements for achieving therapeutic exposure (243, 268). These delivery approaches enhance intestinal absorption, inhibit rapid metabolism, and prolong systemic circulation, thereby providing a mechanistic basis for the observed improvements in clinical outcomes (267, 268). Consequently, the form of the intervention must be considered as critically important as the compound itself when evaluating translational potential.

Another limitation relates to safety nuances that require careful consideration. While plant-derived antioxidants are generally regarded as safe in dietary amounts, some polyphenols may exert pro-oxidant effects under specific conditions, such as high doses in the presence of free transition metal ions (269–271). Moreover, these compounds have the potential to interact with chemotherapy drugs by modulating cytochrome P450 enzymes and drug transporters such as P-glycoprotein (272). Although such risks remain largely theoretical at nutritional doses, they highlight the importance of systematic pharmacokinetic and interaction studies to ensure patient safety in clinical applications.

Despite promising mechanistic foundations and preliminary clinical evidence, significant limitations constrain the clinical application of plant food neuroprotection strategies. The most critical limitation is the scarcity of large-scale, well-controlled clinical trials specifically designed for chemotherapy-induced peripheral neuropathy prevention (2, 163, 164, 273). Current evidence relies primarily on single studies, cross-condition extrapolation, and preclinical models that cannot provide the definitive evidence required for clinical practice guidelines. Methodological challenges include the difficulty of standardizing complex plant food interventions and the natural variability in bioactive compound content across different sources, seasons, and preparation methods. The lack of validated biomarkers for early neuroprotective effects and the reliance on subjective symptom reporting further complicate intervention assessment and optimization.

Future research priorities center on three critical areas. First, large-scale multicenter randomized controlled trials are urgently needed to evaluate plant food interventions in diverse patient populations receiving different chemotherapy regimens. These studies should utilize standardized outcome measures and incorporate biomarker assessments to provide objective evidence of neuroprotective efficacy. Second, mechanistic research should focus on individual variability in plant food metabolism and response, including genetic polymorphisms affecting compound absorption and effectiveness. This research can inform personalized nutrition approaches that optimize interventions based on individual characteristics and treatment protocols. Third, standardization efforts must address intervention reproducibility through development of quality control methods for plant food characterization, establishment of preparation protocols, and creation of practical implementation guidelines that translate research findings into clinical recommendations while maintaining safety standards. The integration of these research priorities with systematic safety evaluations and long-term outcome studies will provide the evidence base necessary for widespread clinical adoption of plant food neuroprotection strategies.

## 7 Conclusion

The Level I evidence for curcumin in pediatric patients is particularly compelling, providing definitive proof-of-concept for plant-based neuroprotection. This comprehensive review demonstrates that plant food-derived antioxidant nutrients represent a promising approach to neuroprotection against chemotherapy-induced neurotoxicity. The mechanistic foundations are well-established, with plant foods providing multi-target protection through antioxidant system activation, anti-inflammatory effects, neurotrophic factor support, and mitochondrial preservation. The clinical evidence, while requiring further validation, provides encouraging proof-of-concept, with curcumin demonstrating the strongest validation and other plant foods showing supportive evidence from related conditions.

The practical advantages of plant food approaches include established safety profiles, widespread accessibility, and cost-effectiveness compared to pharmaceutical alternatives. However, significant limitations constrain clinical implementation, particularly the scarcity of large-scale clinical trials specifically designed for chemotherapy-induced peripheral neuropathy prevention. Future research priorities include large-scale multicenter randomized controlled trials and development of standardized intervention protocols. Plant food-derived antioxidant nutrients are a safe, accessible, and evidence-based strategy that warrants continued investigation as a complement to conventional cancer therapy, with the potential to transform supportive oncology care.
